# Mannose-binding lectin (MBL) and the lectin complement pathway play a role in cutaneous ischemia and reperfusion injury

**DOI:** 10.1515/iss-2020-0017

**Published:** 2020-09-14

**Authors:** Claas-Tido Peck, Sarah Strauß, Gregory L. Stahl, Peter-Maria Vogt, Marc N. Busche

**Affiliations:** Hannover Medical School, Department of Plastic, Aesthetic, Hand, and Reconstructive Surgery, Hannover, Germany; Harvard Medical School, Center for Experimental Therapeutics and Reperfusion Injury, Brigham and Women’s Hospital, Boston, MA, USA; Leverkusen Hospital gGmbH, Department of Plastic and Aesthetic Surgery, Burn Surgery, Leverkusen, Germany

**Keywords:** complement system, IL10, IL13, ischemia-reperfusion injury (I/R), mannose-binding lectin, MBL, skin

## Abstract

**Objectives:**

Cutaneous ischemia/reperfusion (CI/R) injury has shown to play a significant role in chronic wounds such as decubitus ulcers, diabetic foot ulcers, atherosclerotic lesions, and venous stasis wounds. CI/R also plays a role in free tissue transfer in reconstructive microsurgery and has been linked to clinical burn-depth progression after thermal injury. While the role of the complement system has been elucidated in multiple organ systems, evidence is lacking with respect to its role in the skin. Therefore, we evaluated the role of the complement system in CI/R injury.

**Methods:**

Using a single pedicle skin flap mouse model of acute CI/R, we performed CI/R in wild-type (WT) mice and complement knock out (KO) mice, deficient in either C1q (C1q KO; classical pathway inhibition), mannose-binding lectin (MBL null; lectin pathway inhibition) or factor B (H2Bf KO; alternative pathway inhibition). Following 10 h ischemia and 7 days reperfusion, mice were sacrificed, flaps harvested and flap viability assessed via Image J software. The flap necrotic area was expressed as % total flap area. In another group, mice were sacrificed following CI/R with 10 h ischemia and 48 h reperfusion. Two cranial skin flap samples were taken for gene expression analysis of IL1b, IL6, TNFα, ICAM1, VCAM1, IL10, IL13 using real-time polymerase chain reaction (RT-PCR).

**Results:**

Following CI/R, MBL null mice had a statistically significant smaller %necrotic flap area compared to WT mice (10.6 vs. 43.1%; p<0.05) suggesting protection from CI/R. A significantly reduced mean %necrotic flap area was not seen in either C1q KO or H2Bf KO mice relative to WT (22.9 and 31.3 vs. 43.1%; p=0.08 and p=0.244, respectively). There were no statistically significant differences between groups for markers of inflammation (TNFα, ICAM1, VCAM1, IL1b, IL6). In contrast, mRNA levels of IL10, a regulator of inflammation, were significantly increased in the MBL null group (p=0.047).

**Conclusions:**

We demonstrated for the first time a significant role of MBL and the lectin complement pathway in ischemia/reperfusion injury of the skin and a potential role for IL10 in attenuating CI/R injury, as IL10 levels were significantly increased in the tissue from the CI/R-protected MBL null group.

## Introduction

Reperfusion of hypoxic tissue following transient ischemia leads to an immune response of the innate immune system, such as the complement system, against neo-antigens, that have formed on the surface of cells during ischemia [1]. This damage of the reperfused tissue following transient ischemia is known as ischemia-reperfusion (I/R) injury [1].

Cutaneous ischemia/reperfusion (CI/R) injury has been shown to be an important factor in chronic wounds [2], [3]. Chronic wounds, such as pressure ulcers (decubitus ulcers), diabetic foot ulcers, atherosclerotic lesions, and venous stasis wounds [4], are major health problems that affect millions of people worldwide and cause economic burdens on health care systems [3]. In contrast to the well-orchestrated process of normal wound healing, chronic wounds are thought to exhibit a persistent and multifactorial proinflammatory state that delays and prevents healing [5]. Interestingly, repetitive CI/R injury has also been suggested to be a mechanism for clinical burn-depth progression after thermal injury [6]. A third clinical area of interest for CI/R is microsurgery, including free tissue transfer in reconstructive plastic surgery, which involves an obligatory ischemic period until the microanastomoses have been established [7].

**Figure 1:**
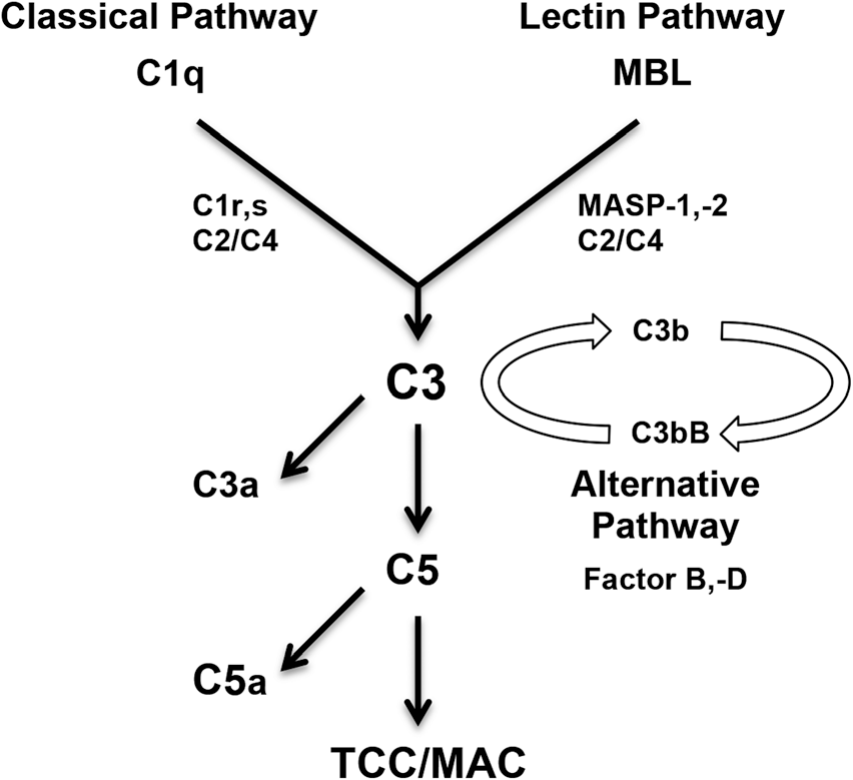
Complement pathways diagram outlining the three complement pathways. MBL = mannose-binding lectin; MASP = MBL-associated serine protease; TCC = terminal complement complex; MAC = membrane attack complex.

Although the complement system has been shown to play a critical role in I/R injury of several organ systems, such as myocardium, muscle, ileum, liver, brain and kidney [8], [9], [10], [11], [12], very limited data exist for the role of the complement system in I/R injury of the skin, the biggest organ. Complement can be activated by at least three separate pathways: classical, alternative and lectin complement pathway (Figure 1). The classical complement pathway is activated following antibody-antigen interactions of natural IgM with neo-antigens [13]. The alternative pathway can be activated by C3b deposition due to classical/lectin pathway activation (i.e., tick-over amplification) and thus enhance classical and lectin pathway activation. Mannose-binding lectin (MBL) and ficolins, carbohydrate recognition subcomponents of the lectin pathway, can also initiate complement activation. MBL is an important part of the immune defense [14], [15] and can play a critical role in inflammatory-mediated induction and tissue injury following I/R [12], [16], [17]. All three pathways merge at C3 and cleave C3 into C3b and C3a by two structurally different, yet functionally equivalent C3 convertases. Cleavage of C3 results in the formation of a C5 convertase and ultimate cleavage of C5 into C5a and C5b. The anaphylatoxin C5a is a potent activator of inflammation and a chemoattractant for inflammatory cell populations, including polymorphonuclear leukocytes [1]. Additionally, C5b can interact with C6, C7, C8, and multiple C9 units and lead to formation of C5b-9 (terminal complement complex or membrane attack complex). C5b-9 can lead to cellular activation or lysis [1].

While inflammation plays an important role in the manifestation of I/R injury [1], [2], [3] the immunomodulatory cytokines IL10 and IL13 have been shown to significantly attenuate I/R injury due to their anti-inflammatory properties *in vivo* [18], [19].

Since the role of the complement system and the different complement pathways has not been elucidated for CI/R, we investigated its role using complement knock out (KO) mice in a skin flap mouse model of CI/R and measured the mRNA expression levels of markers of inflammation (TNFα, ICAM1, VCAM1, IL1b, IL6) and regulators of inflammation (IL10 and IL13) in the tissue following CI/R.

## Materials and methods

### Mice

Animals were bred and kept at the Institution’s Central Animal Facility, were housed under standard conditions, and experiments were carried out in accordance with the guidelines of the German Animal Welfare Act (#09/1781). All animals used in the CI/R experiments were 8- to 12-week old male mice. We obtained C57BL/6J wild type (WT) mice from Jackson Laboratories (Bar Harbor; Maine, USA) via Charles River (Sulzfeld, Germany). Complement KO mice, deficient in either C1q (C1q KO mice; inhibition of the classical pathway), MBL (null mice; inhibition of the lectin pathway) or factor B (H2Bf KO mice; inhibition of the alternative pathway) were backcrossed seven generations in the Center for Experimental Therapeutics and Reperfusion Injury, Brigham and Women’s Hospital, Harvard Medical School, Boston, MA, USA and the Department of Anesthesiology and Intensive Care Therapy, Jena University Hospital, Jena, Germany onto the C57BL/6J background while monitoring the genetic background using microsatellite analysis. After having obtained the complement KO mice from the Jena University Hospital, the identical C57BL/6J background of all complement KO mice was again checked and confirmed by the Institution’s Department of Genetic Diagnostics, Institute for Laboratory Animal Science.

### CI/R experiments

**Figure 2:**
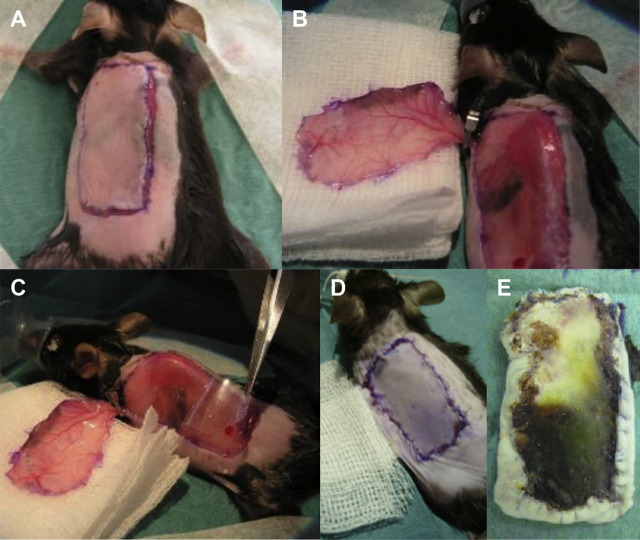
The CI/R model. (A) A 3.5 × 1.5 cm dorsolateral skin flap, located from the midline of the dorsum to the axillary line and from the iliac crest to the subscapular line was raised from caudal to cranial. (B) The two main vascular networks on the undersurface of the flap were defined. The caudal pedicle was cut and the cranial pedicle entering the flap superolaterally from the lateral thoracic artery was preserved and occluded using a 7-mm microclamp. On the downside of the raised flap it is clearly visible, that the most caudal portion of the flap (approximately 0.5 cm) is not perfused by the occluded cranial pedicle, but by the previously cut caudal pedicle. This area was not reperfused following clamp removal after 10 h of ischemia. Therefore, the most distal 0.5 cm of each flap was not included in the Image J evaluation of CI/R-related skin flap necrosis. (C) A medical grade silicon sheet was placed between the muscle and the flap as a barrier to vascular invasion and sutured in place with 7-0 polypropylene. (D) The flap was repositioned and sutured with a continuous 6-0 polypropylene suture to prevent distortion of the vascular pedicles, while interrupted sutures were used at the cranial borders of the flap to provide easy clamp removal following 10 h of ischemia. (E) Typical skin flap necrotic area in a WT mouse following CI/R with 10 h of ischemia and 7 days of reperfusion. CI/R = cutaneous ischemia/reperfusion; WT = wild type.

For the CI/R experiments, we used a previously described single pedicle skin flap mouse model of acute CI/R [20]. Mice were anesthetized by isoflurane. Initial knock-down was performed with 5.0% isoflurane/oxygen and maintained with 1.5–2.0%. Flow rate was at 1 Lmin^−1^. Animals were placed on a heating pad to maintain body temperature at 37 °C during the surgical procedure. The dorsum of each mouse was shaved with an ISIS GT420 electric animal shaver (Aesculap Suhl GmbH, Germany), and residual hair was removed thoroughly with depilatory cream. The surgical area was cleansed and treated with Octenisept (Schuelke & Mayr GmbH, Germany) for disinfection. A 3.5 × 1.5 cm dorsolateral skin flap, located from the midline of the dorsum to the axillary line and from the iliac crest to the subscapular line was raised from caudal to cranial [20]. While raising the flap, the two main vascular networks on the undersurface of the flap, together with the two connecting pedicles, caudally from the deep circumflex iliac artery system and cranially (superolateral) from the lateral thoracic artery were defined. The caudal pedicle was cut and the cranial pedicle entering the flap superolaterally was preserved (Figure 2). A medical grade silicon sheet (Bioplexus, Ventura, CA, USA) was placed between the muscle and the flap as a barrier to vascular invasion and sutured in place with 7-0 Prolene (Ethicon, USA). The cranial vascular pedicle from the lateral thoracic artery was occluded using a 7-mm micro clamp (S&T, Neuhausen, Switzerland). The flap was repositioned and sutured with a continuous 6-0 polypropylene suture to prevent distortion of the vascular pedicles, while interrupted sutures were used at the cranial borders of the flap to provide easy clamp removal. During the ischemia time of 10 h, mice were kept in cages with free access to food and water. After 10 h, the animals were reanesthetized with isoflurane for clamp removal and resuturing (Figure 2). After 7 days of reperfusion, the mice were sacrificed through cervical dislocation under isoflurane anesthesia, the flap was harvested and flap viability was assessed via Image J software (NIH, Bethesda, MD). Another group of mice was treated as described before but sacrificed after 48 h of reperfusion to collect two cranial skin samples of the flap using a 6-mm biopsy punch for RNA extraction and gene expression analysis with real-time polymerase chain reaction (RT-PCR) (GlaxoSmithKline GmbH & Co. KG, Germany). In the sham-operated group, WT mice were treated exactly the same, but without cranial vascular pedicle occlusion with a micro clamp.

In doing surgical I/R experiments, choosing the appropriate ischemia time and subsequent reperfusion time are crucial and dependent upon the animal model and organ system under investigation. When establishing a new model, determining the appropriate time intervals would be first investigated during preliminary model development. Since we used a previously published model for the evaluation of flap viability and successfully replicated the results, we were confident in continuing with the ischemia time of 10 h and a reperfusion time of 7 days. This resulted in skin flap necrosis representing irreversible I/R damage, which can be objectively validated with Image J. However, to ultimately show differential gene expression in response to I/R damage, we had to modify the reperfusion time. Our preliminary experiments demonstrated that reperfusion times exceeding 48 h negatively impacted the quality of isolated RNA to a point where even a stable detection of house-keeping genes was not reliably possible for every mouse strain. As a result, 48 h resulted in a sufficient change in gene expression while maintaining RNA quality.

### Evaluation of flap viability

**Figure 3:**
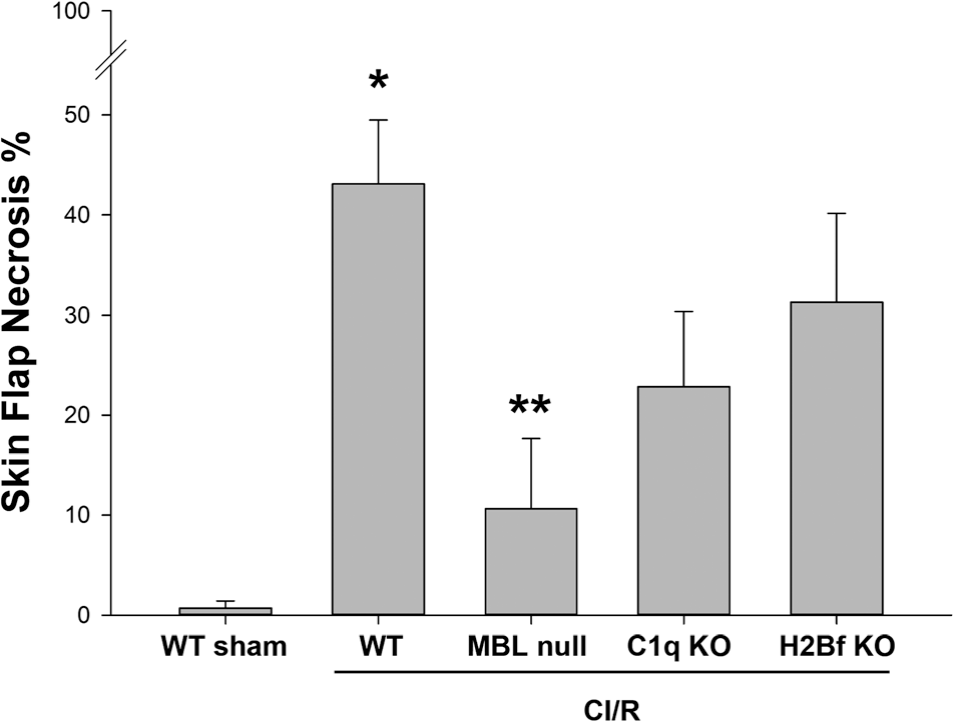
Percentage of skin flap necrotic areas following cutaneous ischemia/reperfusion (CI/R). Following CI/R with 10 h of ischemia and 7 days of reperfusion, mean percentages (average of three measurements per animal per group) of necrotic flap area of the cranial 3.0 × 1.5 cm portion of the flap were determined using Image J. Groups: sham-operated WT mice (WT sham), WT mice undergoing CI/R (WT), MBL null mice undergoing CI/R (MBL null), C1q KO mice undergoing CI/R (C1q KO) and H2Bf KO mice undergoing CI/R (H2Bf KO). All CI/R data are means ± SE of 6–12 animals per group. *p<0.05 compared to sham-operated WT mice (WT sham). **p<0.05 compared to WT mice undergoing CI/R (WT). KO = knock out; WT = wild type.

Following 10 h of ischemia and 7 days of reperfusion, pictures were taken of the freshly harvested flaps and flap viability was assessed by evaluation of pictures via Image J software (NIH, Bethesda, MD). While using the established CI/R model by Tatlidede et al. with a flap size of 3.5 × 1.5 cm in our CI/R experiments [20], we observed, in contrast to that study, the lower 0.5 cm of the flap, in almost all mice, including sham-operated mice, to be completely necrotic. Clearly, this portion of the flap was not perfused from the cranial pedicle, but rather from the caudal pedicle, that was cut while raising the flap. This caudally-based perfusion of the caudal portion of the flap is also visible on the underside of the raised flap (Figure 2). To ensure that necrosis of the flap was due to I/R injury and not due to pure ischemia without subsequent reperfusion, we only included the cranial 3.0 × 1.5 cm of the flap in the Image J evaluation. In future studies using the single pedicle skin flap mouse model by Tatlidede et al. we recommend to decrease the flap size during surgery to the cranial 3.0 × 1.5 cm, making modifications of the Image J evaluation unnecessary. The necrotic area of the cranial 3.0 × 1.5 cm portion of each flap was expressed as a percentage of the total cranial 3.0 × 1.5 cm flap area and is the average of three distinct measurements (Figure 3).

### RT-PCR

Mice were sacrificed following CI/R with 10 h of ischemia and 48 h of reperfusion. After investigating mRNA levels after various reperfusion time periods, 48 h offered the best potential to detect differential expression levels. Two cranial skin samples of the flap were taken for RNA extraction and gene expression analysis using a 6 mm biopsy punch (GlaxoSmithKline GmbH & Co. KG, Germany). Samples were cut in 1 mm thin slices with a ToughCut scissor (Fine Science Tools GmbH, Germany), immediately flash-frozen in liquid nitrogen and transferred to −80 °C. Cutting of the slices was rapid (no more than 5 s) to prevent RNA lysis. Using a biopsy punch at defined cranial skin areas for the sample harvesting ensured that the tissue weight did not differ between the mice and groups. Samples from each individual were pooled and transferred to a mortal filled with liquid nitrogen. A pestle was used to grind the samples. Ground sample was transferred to 1 mL QIAzol (QIAGEN, Germany) for 5 min at room temperature. 0.2 mL chloroform was added and mixed vigorously with a vortexer for 15 s. Samples were kept for 2–3 min at room temperature, followed by a centrifugation step at 12,000 × g for 15 min at 4 °C in a pre-cooled centrifuge. The upper aqueous phase containing the RNA was transferred carefully to a new tube, avoiding contaminations from the middle and lower phases. 0.5 mL isopropanol was added and mixed using a vortexer. A second centrifugation step was performed at 12,000 × g for 10 min at 4 °C to collect the RNA pellet at the bottom of the tube. Supernatant was removed and the pellet was air-dried. Finally, the pellet was resolved in 20 µL RNase-free water. RNA quality was checked using a nanodrop 1000 (ThermoFisher Scientific, Germany). An ethanol precipitation step was used to remove phenol remnants. Two volumes of ice-cold ethanol were added to the tube, followed by 0.25 volume of 10 M ammonium acetate and mixed. The tube was stored for at least 1 h at −20 °C. To recover the RNA, an additional centrifugation step was performed at 12,000 × g for 10 min at 4 °C. The RNA pellet was dissolved again in RNA-free water. A second quality check with the nanodrop showed no phenol remnants. RNA transcription to cDNA with 1 µg RNA each, was performed in a Mastercycler (Eppendorf, Germany) using iScript™ cDNA Synthesis Kit (Bio-Rad, Germany). cDNA was stored at −20 °C for further use. PCRs were performed on an iCycler (Bio-Rad, Germany) using precoated custom RT^2^ Profile PCR arrays (QIAGEN, Germany) according to manufacture protocol with SsoFast EvaGreen Supermix (Bio-Rad, Germany). Triplets of each gene/sample were measured. Precoated plates contained the primers for IL1b, IL6, TNFα, ICAM1, VCAM1, IL10, IL13 and the two reference genes ACTB and TBP. PCR results were normalized and analyzed with the qbase+ software (Biogazelle, Belgium).

### Statistics

Statistical analyses of data were performed using SigmaPlot software for Windows (Systat Software GmbH, Germany). All data were evaluated using one-way analysis of variance (ANOVA) and post hoc analysis using the Student-Newman-Keuls method. All data are expressed as the mean ± SE and differences were considered significant at p<0.05.

## Results

### Evaluation of flap viability

Following CI/R with 10 h of ischemia and 7 days of reperfusion, we evaluated the necrotic area of the cranial 3.0 × 1.5 cm portion of each flap via Image J. WT mice undergoing CI/R demonstrated a significantly greater percentage of necrotic area as a percentage of total flap area compared to sham-operated WT mice (43.1% ± 6.4 vs. 0.7% ± 0.7; p<0.05; Figure 3). MBL null mice undergoing CI/R (inhibition of lectin pathway; Figure 1) had a statistically significant smaller percentage of necrotic flap area compared to WT mice undergoing CI/R (10.6% ± 7.0 vs. 43.1% ± 6.4; p<0.05; Figure 3). Thus, MBL null mice appear to be significantly protected from CI/R.

C1q KO mice, lacking a functional classical complement pathway, demonstrated a reduced mean percentage of necrotic flap area compared to WT mice undergoing CI/R. However, these differences were not statistically significant (22.9% ± 7.6 vs. 43.1% ± 6.4; p=0.08; Figure 3).

H2Bf KO mice (inhibition of alternative pathway; Figure 1) had a slightly, but not statistically significant reduced mean percentage of necrotic flap area compared to WT mice undergoing CI/R (31.3% ± 8.8 vs. 43.1% ± 6.4; p=0.244; Figure 3).

### Real-time polymerase chain reaction (PCR)

**Figure 4:**
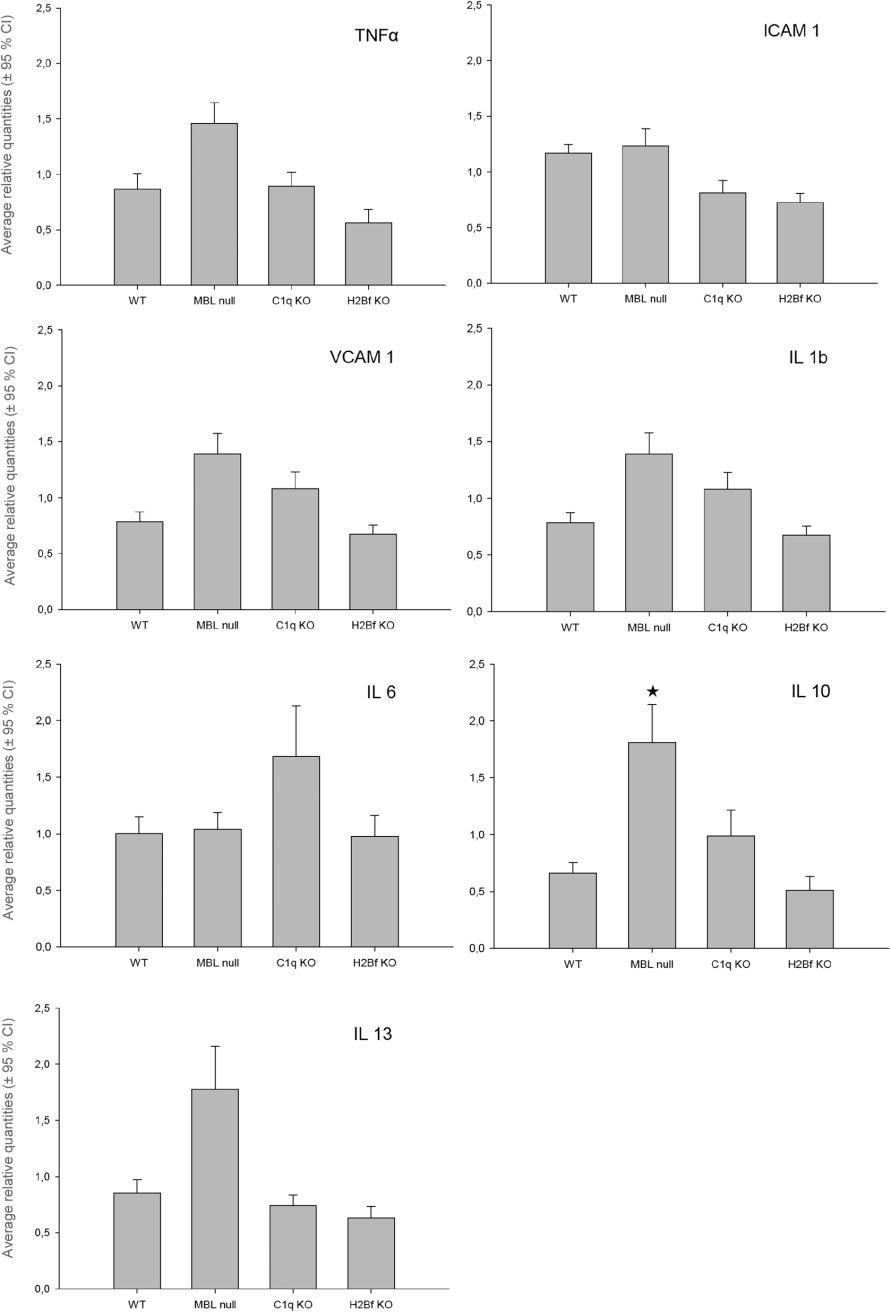
mRNA levels of markers and regulators of inflammation. Following cutaneous ischemia and reperfusion (CI/R) with 10 h of ischemia and 48 h of reperfusion, skin samples were taken from the cranial portion of the flap and evaluated via RT-PCR for expressions of markers of inflammation (TNFα, ICAM1, VCAM1, IL1b, IL6) and regulators of inflammation (IL10 and IL13). Groups: WT mice (WT), MBL null mice (MBL null), C1q KO mice (C1q KO) and H2Bf KO mice (H2Bf KO). All CI/R data are means ± SE of 6–12 animals per group. *p<0.05 compared to all other groups. MBL = mannose-binding lectin; KO = knock out; WT = wild type.

Following CI/R with 10 h of ischemia and 48 h of reperfusion, skin samples were taken from the cranial portion of the flap and evaluated via RT-PCR for gene expression of markers of inflammation (TNFα, ICAM1, VCAM1, IL1b, IL6) and regulators of inflammation (IL10 and IL13). There were no statistically significant differences between groups for the five tested markers of inflammation (TNFα, ICAM1, VCAM1, IL1b, IL6; Figure 4). In contrast, mRNA levels of IL-10, an important regulator of inflammation were significantly increased in the MBL null group, compared to all other groups (p=0.047; Figure 4). For IL13, another important regulator of inflammation with proinflammatory functions, there was a clear trend, albeit not statistically significant (p=0.222; Figure 4), for increased levels of mRNA in the MBL null group.

## Discussion

CI/R has been shown to play an important role in chronic wounds, affecting millions of people worldwide [2], [3], [4], [5]. While the involvement of the complement system has been investigated and demonstrated in I/R injury of many organ systems [8], [9], [10], [11], [12], the role of the complement system in I/R injury of the skin (CI/R) has not been elucidated. Using complement KO mice in a skin flap mouse model of CI/R, we demonstrated for the first time a significant role of MBL and the lectin complement pathway in I/R injury of the skin.

MBL null mice, lacking a functional lectin complement pathway, had a statistically significant reduced mean percentage of necrotic flap area compared to WT mice undergoing CI/R. Thus, MBL and the lectin complement pathway play a significant role in CI/R. In contrast, C1q KO mice, lacking a functional classical complement pathway, demonstrated a clear, albeit nonsignificant trend for a reduced mean percentage of necrotic flap area compared to WT mice undergoing CI/R, suggesting a minor role of the classical complement pathway in CI/R. Inhibition of the alternative complement pathway (H2Bf KO mice) however, resulted in a slight, but not significantly reduced mean percentage of necrotic flap area compared to WT mice undergoing CI/R, suggesting an insignificant role of the alternative complement pathway in CI/R.

The involvement of the three complement pathways in I/R injury appears to vary depending on the affected organ system . The classical pathway appears to play a major role in liver I/R [21] and several studies suggested that natural antibodies and the classical pathway are primarily responsible for gastrointestinal I/R injury [22]. However, there are also data for involvement of the alternative pathway in gastrointestinal I/R injury [10] and more recent work demonstrating that gastrointestinal I/R injury is not dependent on C1q and classical pathway activation but rather on MBL and lectin pathway activation [23]. In renal I/R injury, evidence suggests a role for both, the alternative complement pathway [24], and MBL and the lectin complement pathways [15], [25]. In addition, previously published data in myocardial ischemia-reperfusion (MI/R) injury demonstrated a critical role for MBL and the lectin complement pathway as well [16], [26]. Interestingly, and similar to MI/R injury, our findings for CI/R also demonstrated a critical role for MBL and the lectin complement pathway and only minor roles of the classical and alternative pathways.

To investigate the molecular mechanisms leading to CI/R injury, we evaluated skin samples from the cranial (non-necrotic) portion of the flap following CI/R for mRNA expression levels of markers of inflammation (TNFα, ICAM1, VCAM1, IL1b, IL6) and regulators of inflammation (IL10 and IL13). Interestingly, in our CI/R study there was no significant difference in the mRNA levels of markers of inflammation between groups. However, the effect of the different complement pathways on the levels of IL13 and IL10, regulators of inflammation, seem to play an important role in CI/R injury. IL13 has been shown to suppress monocyte production of proinflammatory cytokines, including IL1, IL6, IL8, TNFα, and MIP-1a *in vitro* [19], [27]. The anti-inflammatory effects of IL13 have been linked to the inhibition of nuclear factor κB (NFκB) and activation of signal transducer and activator of transcription 6 (STAT6) [19], [28], [29]. While the anti-inflammatory effects of IL13 prevent lipopolysaccharide-induced lethality and IgG immune complex-induced lung injury *in vivo* [19], [30], [31], the proinflammatory function of IL13 in conjunction with IL4 play a key role in the pathophysiology of asthma [32], [33]. Inhibiting the biological effects of both IL4 and IL13 with the monoclonal antibody dupilumab has been shown to markedly decrease asthma exacerbations and improve lung function [33]. Interestingly, in liver I/R, administration of IL13 significantly reduced liver neutrophil recruitment, hepatocellular I/R injury and liver edema *in vivo* [19]. As IL13 administration in liver I/R had no effect on liver NFκB activation, but greatly increased the activation of STAT6, the hepatoprotective effects of IL13 in liver I/R are likely a result of STAT6 activation [19].

IL10 is one of the most important immunomodulatory cytokines with anti-inflammatory properties [34]. IL10 can reduce antigen presentation, inhibit T cell activation and limit excessive inflammatory reactions in response to endotoxins, e.g. colitis or endotoxin shock [34]. While it is still not fully understood how IL10 mediates its function on the molecular level, IL10 seems to inhibit downstream events in cytokine regulation, via bcl-3 [34]. Similar to IL13, IL10 has been shown to significantly reduce hepatic neutrophil recruitment, liver edema, and liver I/R injury *in vivo*, likely through the suppression of NFκB activation [18].

In our mouse model of CI/R, mRNA levels of IL10 were significantly increased and IL13 message levels showed a clear, but non-significant increase in the CI/R injury protected MBL null group compared to all other groups. Thus, similar to liver I/R, IL10, and IL13 seem to play a role in preventing cutaneous I/R injury. As IL10 and IL13 were only increased in the MBL null group, lacking a functional lectin complement pathway, and in addition to this group having protection from CI/R injury, MBL and the lectin complement pathway may function by suppressing the expression of IL10 and IL13, immunomodulatory cytokines that can potentially reduce CI/R injury. Thus, CI/R injury may not be dependent only on MBL, but also on the absence and/or lower concentrations of IL10 and IL13. While our data suggest an MBL and lectin complement pathway-mediated IL10 suppression, it is also known that IL10 can inhibit activation of the complement system and complement-mediated neutrophil recruitment and migration [35].

Additional studies are required to further understand the role of MBL and the lectin complement pathway in CI/R. Specifically, how CI/R-related tissue damage is mediated on a molecular level and to elucidate the influence of MBL and the lectin pathway on IL10 levels. Moreover, the molecular mechanisms of how IL10 mediates its protective function against CI/R injury need to be investigated. Similar to liver I/R, where I/R injury is attenuated by IL10 and IL13 suppression of NFκB activation and STAT6 activation, the role of NFκB and STAT6 in CI/R injury is of special interest and should be further investigated.

In conclusion, our data demonstrate for the first time a significant role for MBL and the lectin complement pathway in CI/R and suggest that the immunomodulatory cytokine IL10 can attenuate CI/R in the absence of MBL. Since the skin, in contrary to other organs, is easy to reach with local therapy, our data together with findings from future molecular and clinical studies could ultimately result in topical treatments of chronic wounds, thus reducing CI/R injury and inflammation by blocking MBL or the lectin complement pathway in combination with application of immunomodulatory cytokines such as IL10 and IL13.

## References

[1] Riedemann NC , Ward PA . Complement in ischemia reperfusion injury. Am J Pathol 2003;162:363–7. 10.1016/s0002-9440(10)63830-8. 12547694 PMC1851148

[2] Mustoe T . Understanding chronic wounds: a unifying hypothesis on their pathogenesis and implications for therapy. Am J Surg 2004;187:65–70S. 10.1016/s0002-9610(03)00306-4. 15147994

[3] Sisco M , Liu WR , Kryger ZB , Mustoe TA . Reduced up-regulation of cytoprotective genes in rat cutaneous tissue during the second cycle of ischemia-reperfusion. Wound Repair Regen 2007;15:203–12. 10.1111/j.1524-475x.2007.00206.x. 17352752

[4] Loots MA , Lamme EN , Zeegelaar J , Mekkes JR , Bos JD , Middelkoop E . Differences in cellular infiltrate and extracellular matrix of chronic diabetic and venous ulcers versus acute wounds. J Invest Dermatol 1998;111:850–7. 10.1046/j.1523-1747.1998.00381.x. 9804349

[5] Stojadinovic A , Carlson JW , Schultz GS , Davis TA , Elster EA . Topical advances in wound care. Gynecol Oncol 2008;111(2 Suppl):S70–80. 10.1016/j.ygyno.2008.07.042. 18793796

[6] Jaskille AD , Jeng JC , Sokolich JC , Lunsford P , Jordan MH . Repetitive ischemia-reperfusion injury: a plausible mechanism for documented clinical burn-depth progression after thermal injury. J Burn Care Res 2007;28:13–20. 10.1097/bcr.0b013e31802cb82c. 17211195

[7] Siemionow M , Arslan E . Ischemia/reperfusion injury: a review in relation to free tissue transfers. Microsurgery 2004;24:468–75. 10.1002/micr.20060. 15378577

[8] Chan RK , Ibrahim SI , Takahashi K , Kwon E , McCormack M , Ezekowitz A , . The differing roles of the classical and mannose-binding lectin complement pathways in the events following skeletal muscle ischemia-reperfusion. J Immunol 2006;177:8080–5. 10.4049/jimmunol.177.11.8080. 17114482

[9] Costa C , Zhao L , Shen Y , Su X , Hao L , Colgan SP , . Role of complement component C5 in cerebral ischemia/reperfusion injury. Brain Res 2006;1100:142–51. 10.1016/j.brainres.2006.05.029. 16780818

[10] Stahl GL , Xu Y , Hao L , Miller M , Buras JA , Fung M , . Role for the alternative complement pathway in ischemia/reperfusion injury. Am J Pathol 2003;162:449–55. 10.1016/s0002-9440(10)63839-4. 12547703 PMC1851150

[11] Wada K , Montalto MC , Stahl GL . Inhibition of complement C5 reduces local and remote organ injury after intestinal ischemia/reperfusion in the rat. Gastroenterology 2001;120:126–12. 10.1053/gast.2001.20873. 11208721

[12] Walsh MC , Bourcier T , Takahashi K , Shi L , Busche MN , Rother RP , . Mannose-binding lectin is a regulator of inflammation that accompanies myocardial ischemia and reperfusion injury. J Immunol 2005;175:541–6. 10.4049/jimmunol.175.1.541. 15972690

[13] Zhang M , Austen WG , Chiu I , Alicot EM , Hung R , Ma M , . Identification of a specific self-reactive IgM antibody that initiates intestinal ischemia/reperfusion injury. Proc Natl Acad Sci U S A 2004;101:3886–91. 10.1073/pnas.0400347101. 14999103 PMC374339

[14] Holmskov U , Thiel S , Jensenius JC . Collections and ficolins: humoral lectins of the innate immune defense. Annu Rev Immunol 2003;21:547–78. 10.1146/annurev.immunol.21.120601.140954. 12524383

[15] Moller-Kristensen M , Wang W , Ruseva M , Thiel S , Nielsen S , Takahashi K , . Mannan-binding lectin recognizes structures on ischaemic reperfused mouse kidneys and is implicated in tissue injury. Scand J Immunol 2005;61:426–34. 10.1111/j.1365-3083.2005.01591.x. 15882434

[16] Busche MN , Walsh MC , McMullen ME , Guikema BJ , Stahl GL . Mannose-binding lectin plays a critical role in myocardial ischaemia and reperfusion injury in a mouse model of diabetes. Diabetologia 2008;51:1544–51. 10.1007/s00125-008-1044-6. 18493734 PMC2542900

[17] Collard CD , Vakeva A , Morrissey MA , Agah A , Rollins SA , Reenstra WR , . Complement activation after oxidative stress: role of the lectin complement pathway. Am J Pathol 2000;156:1549–56. 10.1016/s0002-9440(10)65026-2. 10793066 PMC1876913

[18] Yoshidome H , Kato A , Edwards MJ , Lentsch AB . Interleukin-10 suppresses hepatic ischemia/reperfusion injury in mice: implications of a central role for nuclear factor κB. Hepatology 1999;30:203–8. 10.1002/hep.510300120. 10385657

[19] Yoshidome H , Kato A , Miyazaki M , Edwards MJ , Lentsch AB . IL-13 activates STAT6 and inhibits liver injury induced by ischemia/reperfusion. Am J Pathol 1999;155:1059–64. 10.1016/s0002-9440(10)65208-x. 10514388 PMC1867010

[20] Tatlidede S , McCormack MC , Eberlin KR , Nguyen JT , Randolph MA , Austen WG . A novel murine island skin flap for ischemic preconditioning. J Surg Res 2009;154:112–7. 10.1016/j.jss.2008.05.029. 19101697

[21] Heijnen BH , Straatsburg IH , Padilla ND , Van Mierlo GJ , Hack CE , Van Gulik TM . Inhibition of classical complement activation attenuates liver ischaemia and reperfusion injury in a rat model. Clin Exp Immunol 2006;143:15–23. 10.1111/j.1365-2249.2005.02958.x. 16367929 PMC1809558

[22] Fleming SD , Shea-Donohue T , Guthridge JM , Kulik L , Waldschmidt TJ , Gipson MG , . Mice deficient in complement receptors 1 and 2 lack a tissue injury-inducing subset of the natural antibody repertoire. J Immunol 2002;169:2126–33. 10.4049/jimmunol.169.4.2126. 12165541

[23] Hart ML , Ceonzo KA , Shaffer LA , Takahashi K , Rother RP , Reenstra WR , . Gastrointestinal ischemia-reperfusion injury is lectin complement pathway dependent without involving C1q. J Immunol 2005;174:6373–80. 10.4049/jimmunol.174.10.6373. 15879138

[24] Zhou W , Farrar CA , Abe K , Pratt JR , Marsh JE , Wang Y , . Predominant role for C5b-9 in renal ischemia/reperfusion injury. J Clin Invest 2000;105:1363–71. 10.1172/jci8621. 10811844 PMC315463

[25] de Vries B , Walter SJ , Peutz-Kootstra CJ , Wolfs TG , van Heurn LW , Buurman WA . The mannose-binding lectin-pathway is involved in complement activation in the course of renal ischemia-reperfusion injury. Am J Pathol 2004;165:1677–88. 10.1016/s0002-9440(10)63424-4. 15509537 PMC1618654

[26] Busche MN , Pavlov V , Takahashi K , Stahl GL . Myocardial ischemia and reperfusion injury is dependent on both IgM and mannose-binding lectin. Am J Physiol Heart Circ Physiol 2009;297:H1853–9. 10.1152/ajpheart.00049.2009. 19749170 PMC2781373

[27] de Waal Malefyt R , Figdor CG , Huijbens R , Mohan-Peterson S , Bennett B , Culpepper J , . Effects of IL-13 on phenotype, cytokine production, and cytotoxic function of human monocytes. Comparison with IL-4 and modulation by IFN-gamma or IL-10. J Immunol 1993;151:6370–81. 7902377

[28] Izuhara K , Heike T , Otsuka T , Yamaoka K , Mayumi M , Imamura T , . Signal transduction pathway of interleukin-4 and interleukin-13 in human B cells derived from X-linked severe combined immunodeficiency patients. J Biol Chem 1996;271:619–22. 10.1074/jbc.271.2.619. 8557662

[29] Takeda K , Kamanaka M , Tanaka T , Kishimoto T , Akira S . Impaired IL-13-mediated functions of macrophages in STAT6-deficient mice. J Immunol 1996;157:3220–2. 8871614

[30] Muchamuel T , Menon S , Pisacane P , Howard MC , Cockayne DA . IL-13 protects mice from lipopolysaccharide-induced lethal endotoxemia: correlation with down-modulation of TNF-alpha, IFN-gamma, and IL-12 production. J Immunol 1997;158:2898–903. 9058827

[31] Mulligan MS , Warner RL , Foreback JL , Shanley TP , Ward PA . Protective effects of IL-4, IL-10, IL-12, and IL-13 in IgG immune complex-induced lung injury: role of endogenous IL-12. J Immunol 1997;159:3483–9. 9317147

[32] Corren J . Role of interleukin-13 in asthma. Curr Allergy Asthma Rep 2013;13:415–20. 10.1007/s11882-013-0373-9. 24026573

[33] Vatrella A , Fabozzi I , Calabrese C , Maselli R , Pelaia G . Dupilumab: a novel treatment for asthma. J Asthma Allergy 2014;7:123–30. 10.2147/jaa.s52387. 25214796 PMC4159398

[34] Grutz G . New insights into the molecular mechanism of interleukin-10-mediated immunosuppression. J Leukoc Biol 2005;77:3–15. 10.1189/jlb.0904484. 15522916

[35] Kulkarni U , Karsten CM , Kohler T , Hammerschmidt S , Bommert K , Tiburzy B , . IL-10 mediates plasmacytosis-associated immunodeficiency by inhibiting complement-mediated neutrophil migration. J Allergy Clin Immunol 2016;137:1487–97 e6. 10.1016/j.jaci.2015.10.018. 26653800

