# Supplementary material for: Mannose-binding lectin (MBL) and the lectin complement pathway play a role in cutaneous ischemia and reperfusion injury
Source: Innov Surg Sci. 2020 Sep 14;5(1-2):43–51. doi: 10.1515/iss-2020-0017 (PMC7798300; doi:10.1515/iss-2020-0017)
Supplement: Supplementary file 1 [file iss-5-20200017-s001.pdf]

Reviewer Assessment

Marc Busche et al.: Mannose-binding lectin (MBL) and the lectin complement pathway play a role in cutaneous ischemia and reperfusion injury

Reviewers' Comments to Original Submission

Reviewer 1: anonymous

Date received: 01-Jun-2020
Reviewer recommendation: **Return to author for minor modifications**
Reviewer overall scoring: **High**

Assessment Form scores: 5 = High/Yes; 3 = Medium/Adequate; 1 = Low

Is the subject area appropriate for the journal	5				
Does the title clearly reflect the paper's content?	5				
Does the abstract clearly reflect the paper's content	5				
Do the keywords clearly reflect the paper's content?	5				
Does the introduction present the problem clearly?		4			
Are the results/ conclusions justified?		4			
How comprehensive and up-to-date is the subject matter presented?	5				
How adequate is the data presentation?	5				
Are units and terminology used correctly?	5				
Is the number of cases adequate?	5				
Are the experimental methods/ clinical studies adequate?	5				
Is the length appropriate in relation to the content?		4			
Does the reader get new insights from the article?	5				
Please rate the practical significance.		4			
Please rate the accuracy of methods.	5				
Please rate the statistical evaluation and quality control.	5				
Please rate the appropriateness of the figures and tables.		4			
Please rate the appropriateness of the references.		4			
Please evaluate the writing style and use of language.	5				
Please judge the overall scientific quality of the manuscript.		4			
Are the methods used worthy of reproduction in greater deal?	yes				

Comments to author: The authors present an experimental animal study on cutaneous ischemia reperfusion injury in a defined skin flap. Data from wild type mice were compared with three different KO mice with inhibition of the lectin pathway,

classic complement or the alternative complement pathway. Size of flap necrosis and inflammatory parameters in the flap tissue were analysed.
The authors utilised a complex surgical study design. This mouse model has previously been described and is an accepted model for similar studies on ischemia reperfusion. In addition to macroscopic observations the authors add further data on possible downstream events possibly explaining the observed results.
While solely the MBL KO mouse showed a significant decrease in necrosis, there was also a trend for improved outcome both other groups (C1qKO and H2Bf KO). However this does not decrease the value of the investigation and subsequent manuscript.
The manuscript is well written and structured, the material and methods section is precise and the discussion is sufficient.
My single point of critic is the timeline for the different procedures. The authors should state why 10 hrs of ischemia, 7 days of observation for the clinical data and 48 hrs for the molecular experiments were chosen. The authors should discuss and add a section on this topic allowing further researches to wisely choose time points in a similar experimental approach.

Reviewer 2: anonymous

Date received: 03-Jul-2020
Reviewer recommendation: **Return to author for minor modifications**
Reviewer overall scoring: **Excellent**

Assessment Form scores: 5 = High/Yes; 3 = Medium/Adequate; 1 = Low

Is the subject area appropriate for the journal	5				
Does the title clearly reflect the paper's content?		4			
Does the abstract clearly reflect the paper's content	5				
Do the keywords clearly reflect the paper's content?	5				
Does the introduction present the problem clearly?	5				
Are the results/ conclusions justified?	5				
How comprehensive and up-to-date is the subject matter presented?	5				
How adequate is the data presentation?	5				
Are units and terminology used correctly?	5				
Is the number of cases adequate?	5				
Are the experimental methods/ clinical studies adequate?	5				
Is the length appropriate in relation to the content?	5				
Does the reader get new insights from the article?	5				
Please rate the practical significance.	5				
Please rate the accuracy of methods.	5				
Please rate the statistical evaluation and quality control.	5				
Please rate the appropriateness of the figures and tables.	5				
Please rate the appropriateness of the references.	5				
Please evaluate the writing style and use of language.	5				
Please judge the overall scientific quality of the manuscript.	5				
Are the methods used worthy of reproduction in greater deal?	yes				

Comments to author: The authors present a nice study concerning the until now unknown effects of the complement system on cutaneous I/R in a mouse skin flap model. The study is interesting and the start of ongoing research. I do only have minor concerns before publication, which have to be addressed.
Minor concerns

Page 3 line 55: please correct „Thec“ to „The“
Page 6 line 56/57: Which animals were used in the Sham group? Also the KO mice? Did you do the KO mice on your own?
Page 7 line 25 ff: Why haven´t you reduced the size of the flap then? Necrosis can also induce complement in the surrounding tissue.
Page 11 line 14/15: please delete the organs, already mentioned in the introduction section.
Title: I would recommend to somehow decrease the „critical“ in the title, as long as it is not clear up to now, how MBL is working and if MBL is the one and only factor in this scenario.

Authors' Response to Reviewer Comments

Date received: 06-Jul-2020

Response to reviewer 1

We We thank the Reviewer for this helpful comment. A section discussing the time points of I/R has been added to the "Materials and Methods" part of the manuscript:

"In doing surgical I/R experiments, choosing the appropriate ischemia time and subsequent reperfusion time are crucial and dependent upon the animal model and organ system under investigation. When establishing a new model, determining the appropriate time intervals would be first investigated during preliminary model development. Since we used a previously published model for the evaluation of flap viability and successfully replicated the results, we were confident in continuing with the ischemia time of 10 h and a reperfusion time of 7 days. This resulted in skin flap necrosis representing irreversible I/R damage, which can be objectively validated with ImageJ. However, to ultimately show differential gene expression in response to I/R damage, we had to modify the reperfusion time. Our preliminary experiments demonstrated that reperfusion times exceeding 48 h negativly impacted the quality of isolated RNA to a point where even a stable detection of house keeping genes was not reliably possible for every mouse strain. As a result, 48 h resulted in a sufficient change in gene expression while maintaining RNA quality."

Response to reviewer 2

Page 3 line 55: please correct „Thec“ to „The“

We thank the author for finding this spelling mistake which has been corrected.

Page 6 line 56/57: Which animals were used in the Sham group? Also the KO mice? Did you do the KO mice on your own?

We used WT mice in the sham group as mentioned in Figure 3. We agree with the Reviewer that we forgot to mention this in the „Materials and Methods“ section of the manuscript. We have corrected this and added this information in the „Materials and Methods“ section of the manuscript as requested by the Reviewer on page 6, line 56/57.

"In the sham-operated group, WT mice were treated exactly the same, but without cranial vascular pedicle occlusion with a micro clamp."

The purpose of a sham-operated group of animals, in which the surgical procedure is exactly reproduced as in the experimental group without the treatment under study (in our case I/R via clamping of the vascular pedicle of the flap) is to demonstrate, that the observed effects are not merely a consequence of the surgery itself, but of the occlusion of the flap's pedicle (treatment under study) and thus truly related to I/R injury. As this can be demonstrated in the WT group, a separate sham group for each type of used complement KO-mice is not necessary, reducing the total number of required animals for the study in accordance with the guidelines of the German Animal Welfare Act (#09/1781).

Page 7 line 25 ff: Why haven´t you reduced the size of the flap then? Necrosis can also induce complement in the surrounding tissue.

This is a very good point by the Reviewer and we agree with the Reviewer, that necrosis can induce complement in the surrounding tissue. However, when we realized, that the lower 0.5 cm of the flap were completely necrotic in almost all mice, a good amount of experimental surgeries had already been performed using the flap size of the originally described model by Tatlidede et al. of 3.5 x 1.5 cm, including mice from all three groups of complement KO mice. To prevent repeating all performed surgeries up to this point and to limit the total amount of required animals for the study (taking the Institution's guidelines and the guidelines of the German Animal Welfare Act into consideration), we decided to keep the flap size of the original model for the rest of the surgeries. Therefore, we rather reduced the Image J evaluated area of each flap to the cranial 3.0 x 1.5 cm. However, for future experiments, a flap size in the surgical model of 3.0 x 1.5 cm instead of the previously described 3.5 x 1.5 cm would be advisable. We have therefore added the following sentence on page 7 line 25 ff: "In future studies using the single pedicle skin flap mouse model by Tatlidede et al., we recommend to decrease the flap size during surgery to the cranial 3.0 x 1.5 cm, making modifications of the Image J evaluation unnecessary."

Page 11 line 14/15: please delete the organs, already mentioned in the introduction section.

The listed organs ("...such as myocardium, skeletal muscle, ileum, liver, brain and kidney...") have been deleted in the mentioned sentence in line 14/15 on the first page of the discussion.

Title: I would recommend to somehow decrease the „critical" in the title, as long as it is not clear up to now, how MBL is working and if MBL is the one and only factor in this scenario.

Although we have demonstrated a statistically significant role for MBL and the lectin complement pathway in CI/R, we agree with the Reviewer, that as mentioned in the "Discussion", further studies are required to fully understand the role of MBL and the lectin complement pathway in CI/R and how CI/R-related tissue damage is mediated on a molecular level. We have therefore changed the title accordingly and have deleted „critical", thus attenuating the statement of the title as requested by the Reviewer.

Reviewers' Comments to Revised Submission

Reviewer 1: anonymous

Date received: 07-Jul-2020
Reviewer recommendation: **Accept in present form**
Reviewer overall scoring: **High**

Assessment Form scores: 5 = High/Yes; 3 = Medium/Adequate; 1 = Low

Is the subject area appropriate for the journal	5				
Does the title clearly reflect the paper's content?		4			
Does the abstract clearly reflect the paper's content	5				
Do the keywords clearly reflect the paper's content?	5				
Does the introduction present the problem clearly?		4			
Are the results/ conclusions justified?		4			

How comprehensive and up-to-date is the subject matter presented?	5				
How adequate is the data presentation?	5				
Are units and terminology used correctly?	5				
Is the number of cases adequate?	5				
Are the experimental methods/ clinical studies adequate?		4			
Is the length appropriate in relation to the content?	5				
Does the reader get new insights from the article?		4			
Please rate the practical significance.		4			
Please rate the accuracy of methods.	5				
Please rate the statistical evaluation and quality control.	5				
Please rate the appropriateness of the figures and tables.		4			
Please rate the appropriateness of the references.		4			
Please evaluate the writing style and use of language.	5				
Please judge the overall scientific quality of the manuscript.		4			
Are the methods used worthy of reproduction in greater deal?	yes				

Comments to author: The authors present a revised manuscript and have addressed my queries. Following the given information future scientists will now be able to continue this line of research.

Reviewer 2: anonymous

Date received: 07-Jul-2020
Reviewer recommendation: **Accept in present form**
Reviewer overall scoring: **Excellent**

Assessment Form scores: 5 = High/Yes; 3 = Medium/Adequate; 1 = Low

Is the subject area appropriate for the journal	5				
Does the title clearly reflect the paper's content?	5				
Does the abstract clearly reflect the paper's content	5				
Do the keywords clearly reflect the paper's content?	5				
Does the introduction present the problem clearly?	5				
Are the results/ conclusions justified?	5				
How comprehensive and up-to-date is the subject matter presented?	5				
How adequate is the data presentation?	5				
Are units and terminology used correctly?	5				
Is the number of cases adequate?	5				
Are the experimental methods/ clinical studies adequate?	5				
Is the length appropriate in relation to the content?	5				
Does the reader get new insights from the article?	5				
Please rate the practical significance.	5				
Please rate the accuracy of methods.	5				
Please rate the statistical evaluation and quality control.	5				
Please rate the appropriateness of the figures and tables.	5				
Please rate the appropriateness of the references.	5				

Please evaluate the writing style and use of language.	5				
Please judge the overall scientific quality of the manuscript.	5				
Are the methods used worthy of reproduction in greater deal?	yes				

Comments to author: All points have been addressed. I accept the manuscript now for publication.
